# Cloning and Functional Analysis of Three Diacylglycerol Acyltransferase Genes from Peanut (*Arachis hypogaea* L.)

**DOI:** 10.1371/journal.pone.0105834

**Published:** 2014-09-02

**Authors:** Xiaoyuan Chi, Ruibo Hu, Xiaowen Zhang, Mingna Chen, Na Chen, Lijuan Pan, Tong Wang, Mian Wang, Zhen Yang, Quanfu Wang, Shanlin Yu

**Affiliations:** 1 Shandong Peanut Research Institute, Qingdao, P R China; 2 Key Laboratory of Biology and Genetic Improvement of Oil Crops, Ministry of Agriculture, Oil Crops Research Institute, Chinese Academy of Agricultural Sciences, Wuhan, P R China; 3 Qingdao Institute of BioEnergy and Bioprocess Technology, Chinese Academy of Sciences (QIBEBT-CAS), Qingdao, P R China; 4 Yellow Sea Fisheries Research Institute, Chinese Academy of Fishery Sciences, Qingdao, P R China; 5 School of Marine and Technology, Harbin Institute of Technology, Weihai, P R China; Wuhan University, China

## Abstract

Diacylglycerol acyltransferase (DGAT) catalyzes the final and only committed acylation step in the synthesis of triacylglycerols. In this study, three novel *AhDGATs* genes were identified and isolated from peanut. Quantitative real-time RT-PCR analysis indicated that the *AhDGAT1-2* transcript was more abundant in roots, seeds, and cotyledons, whereas the transcript abundances of *AhDGAT1-1* and *AhDGAT3-3* were higher in flowers than in the other tissues examined. During seed development, transcript levels of *AhDGAT1-1* remained relatively low during the initial developmental stage but increased gradually during later stages, peaking at 50 days after pegging (DAP). Levels of *AhDGAT1-2* transcripts were higher at 10 and 60 DAPs and much lower during other stages, whereas *AhDGAT3-3* showed higher expression levels at 20 and 50 DAPs. In addition, *AhDGAT* transcripts were differentially expressed following exposure to abiotic stresses or abscisic acid. The activity of the three *AhDGAT* genes was confirmed by heterologous expression in a *Saccharomyces cerevisiae* TAG-deficient quadruple mutant. The recombinant yeasts restored lipid body formation and TAG biosynthesis, and preferentially incorporated unsaturated C18 fatty acids into lipids. The present study provides significant information useful in modifying the oil deposition of peanut through molecular breeding.

## Introduction

Plant lipids are composed of a wide variety of fatty acids and their derivatives, including glycerolipids, lipid polyesters, sterols, and so on. Plant lipids are involved in a diverse range of metabolic reactions and play important physiological roles in plant development and stress regulation, as major components of cellular membranes, storage reserves, extracellular protective layers, and signaling molecules [1]. The biosynthesis of these different types of lipids is controlled by a complex network of genes and proteins.

Triacylglycerols (triglycerides; TAGs), as the major storage forms of energy, have essential functions in multiple physiological processes. In plants, TAGs are crucial for seed oil accumulation, germination, and seedling development [2], [3]. TAGs are synthesized by the enzymes of the Kennedy pathway, which sequentially transfer acyl chains from acyl-CoAs to the sn-1, -2 and -3 positions of a glycerol backbone [4]. Diacylglycerol acyltransferase (DGAT) catalyzes the final acylation of the pathway, which is the only step unique to TAG synthesis. DGAT enzyme activity is encoded by at least four classes of genes in eukaryotes. The type 1 class of DGAT enzymes (DGAT1), which show high sequence homology to mammalian acyl-CoA/cholesterol acyltransferases (ACAT; EC 2.3.1.26), was initially described in mouse and subsequently in several plant species [5], [6]. DGAT1 is structurally related to the ACATs, with the divergence in its amino acid sequence conferring its substrate specificity to DAG. Both enzymes belong to a large family of membrane-bound O-acyltransferases (MBOAT) [7]. The type 2 class of DGAT enzymes (DGAT2) that shares sequence similarity with acyl-CoA: monoacylglycerol acyltransferases (MGAT; EC 2.3.1.22) and acyl-CoA wax alcohol acyltransferases (AWAT; EC 2.3.1.75) was also reported in fungi, *Caenorhabditis elegans*, humans and some plant species [8]. DGAT1 and DGAT2 exhibit no sequence homology with each other. Furthermore, DGAT1 proteins are larger than DGAT2 and possess six to nine transmembrane domains compared to the one or two predicted in DGAT2 [1]. The type 3 class of DGAT enzymes (DGAT3) is a soluble cytosolic protein, which was initially isolated from developing peanut cotyledons through protein purification [9] and was recently isolated from *Arabidopsis* [10]. The type 4 class of DGAT enzymes is represented by the bifunctional DGAT/wax ester synthase (ADP1) from *Acinetobacter calcoaceticus* [11]. Homologs of ADP1 have also been characterized in *Petunia* [12] and *Arabidopsis* [13]. In *Euonymus alatus*, another type of DGAT was recently identified that is responsible for the synthesis of 3-acetyl-1,2-diacyl-sn-glycerols (acTAG), an unusual triacylglycerol [14]. The specific functions of these DGAT enzymes in TAG biosynthesis in oilseeds or lipid bodies vary in different organisms and even in different tissues within the same species [8], [15].

A number of studies have focused on *DGATs* because of their important roles in TAG synthesis. Overexpression studies of these genes have been performed in insect, mammalian, yeast cells, algae, and plants in the past. For example, overexpression of the *Arabidopsis DGAT1* gene in tobacco and yeast greatly enhanced the TAG content of the transformed lines [16], [17], [18]. Interestingly, *Ricinus communis DGAT2* (*RcDGAT2*) has a strong preference for hydroxyl fatty acids containing DAG substrates, and their levels increased from 17% to nearly 30% when *RcDGAT2* was expressed in *Arabidopsis* [19]. Overexpression of a codon-optimized version of *Umbelopsis ramanniana DGAT2A* in soybean seed resulted in an absolute increase in oil of 1.5% (by weight) in the mature seed [20]. *Arabidopsis* (*AtDGAT1*) and *Brassica napus DGAT1s* (*BnDGAT1*) were overexpressed in canola under the control of seed specific promoters. *AtDGAT1* was inserted into *B. napus* cultivar ‘Quantum’, whereas *BnDGAT1* was introduced into the *B. napus* double haploid breeding line DH12075. Both sets of transgenic plants exhibited increased seed oil contents, ranging from 2.5% to 7% of dried mass on an absolute basis, in greenhouse experiments and field trials [21]. Expression of *EaDAcT* under the control of a seed-specific promoter in *Arabidopsis* resulted in 3-acetyl-1,2-diacyl-snglycerols (acTAGs) representing 40% (mol) of total TAGs in the seed oil [14]. Coexpression of an epoxygenase from *Stokesia laevis*, *SlEPX,* and *VgDGAT1* or *VgDGAT2* from *Vernonia galamensis* greatly increased accumulation of vernolic acid both in petunia leaves and soybean somatic embryos [22]. Overexpression of *PtDGAT2* in *Phaeodactylum tricornutum* stimulated more oil bodies, and the neutral lipid content increased by 35%. The fatty acid composition showed a significant increase in the proportion of polyunsaturated fatty acids [23]. Expression of *Chlamydomonas reinhardtii DGTT2* in *Arabidopsis* increased the leaf TAG content, with some molecular species containing very long chain fatty acids [24].

Peanut (*Arachis hypogaea* L.) is an allotetraploid species (2n = 4x = 40, AABB) and one of the five most important oilseed crops worldwide. It is grown extensively in tropical, subtropical, and temperate climates [25]. The peanut seed comprises around 50% oil, of which approximately 80% consists of oleic (36–67%) and linoleic (15–43%) acids [26]. Several molecular studies of lipid biosynthesis in peanut have been reported in recent years [9], [27], [28]. In the present study, we isolated three novel *DGAT* genes from peanut. The expression patterns of these genes were investigated in different tissues and at different stages of seed development. The expression of *DGAT* genes’ response to abiotic stress and abscisic acid (ABA) was also analyzed. Additionally, their functions were confirmed by heterologous expression in the yeast *Saccharomyces cerevisiae* TAG-deficient mutant. Our results indicate that these three genes are strong candidates for modifying lipid biosynthesis in peanut seeds.

## Results

### Isolation of DGAT genes from peanut

Three genes that likely encode DGAT proteins were found using Bioedit software. They were cloned and designated as *AhDGAT1-1*, *AhDGAT1-2*, and *AhDGAT*3-*3* according to the homologous genes identified in *Arabidopsis*. Among the three genes, two genes have the complete open reading frames in the peanut cDNA library and were cloned by conventional RT-PCR; however, one gene was cloned using the rapid amplification of cDNA ends (RACE) method. The open reading frames of *AhDGAT1-1*, *AhDGAT1-2*, and *AhDGAT*3-*3* were 1,539 bp, 1,581 bp, and 1,023 bp in length, encoding 512, 526, and 340 amino acids, respectively (Table 1). The sequence information for the three genes was submitted to Genbank, with the identification numbers KC736068, KC736069, and KC736067, respectively.

**Table 1:** Diacylglycerol acyltransferase genes in peanut.

Protein	Accession	Len (aa)	ORF (bp)	5′ upstream region (bp)	3′ downstream region (bp)	Molecular mass (kDa)	PI
DGAT1-1	KC736068	512	1,539	277	254	58.6036	8.83
DGAT1-2	KC736069	526	1,581	229	743	60.6598	8.94
DGAT3-3	KC736067	340	1,023	96	258	36.911	8.17

A search using NCBI BLAST revealed that the three DGAT proteins have high sequence similarities with DGATs in *Arabidopsis* and soybean (Fig. S1). AhDGAT1-1 shares 63.6%, 76.0%, 75.5%, and 69.2% amino acid sequence identities with AtDGAT1 (AT2G19450), GmDGAT1-1 (Glyma13G106100), GmDGAT1-2 (Glyma17G053300), and GmDGAT1-3 (Glyma09G065300), respectively. AhDGAT1-2 shows 60.4%, 63.8%, 64.1%, and 65.4% similarity with AtDGAT1 (AT2G19450), GmDGAT1-1 (Glyma13G106100), GmDGAT1-2 (Glyma17G053300), and GmDGAT1-3 (Glyma09G065300), respectively. The AhDGAT3-3 protein is most similar to AhDGAT3-1 (90.3%) and AhDGAT3-2 (85.0%). AhDGAT3-3 shares 28.1%, 46.7%, and 45.8% amino acid sequence identities with AtDGAT3 (AT1G48300), GmDGAT3-1 (Glyma13G118300), and GmDGAT3-2 (Glyma17G041600), respectively.

**Figure 1:**
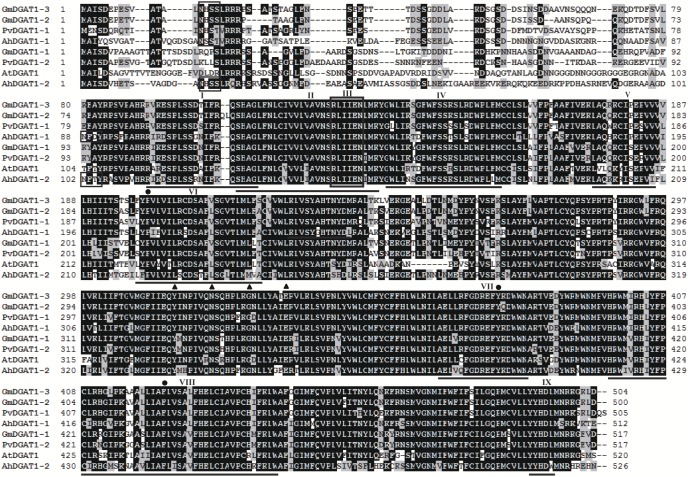
Homology comparison of the amino acid sequences of AhDGAT1 with DGAT1s from other plant species. Identical amino acid residues are highlighted in black. A putative acyl-CoA binding motif is underlined and designated as block ‘I’. The AS11 tandem repeat is underlined and designated as block ‘II’. The putative catalytic active site is underlined and designated as block ‘III’. The phosphopantetheine attachment site is underlined and designated as block ‘IV’. The SnRK1 target site is designated as block ‘V’. The putative thiolase acyl-enzyme intermediate signature is underlined and designated as block ‘VI’; the dot shows the invariant proline. The putative fatty acid protein signature is underlined and designated as block ‘VII’; the dot shows the tyrosine phosphorylation site. The DAG/phorbol ester binding signature motif is underlined and designated as block ‘VIII’; the dot shows the conserved phenylalanine. The putative C-terminal ER retrieval motifs is underlined and designated as block ‘IX’. The N-glycosylation sites are boxed. Amino acids denoted with triangle represent leucine (L) residues forma putative leucine zipper motif.

**Figure 2:**
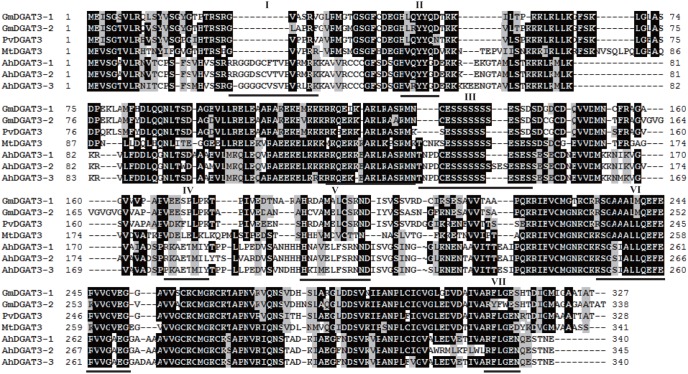
Homology comparison of the amino acid sequences of AhDGAT3 with DGAT3s from other plant species. Identical amino acid residues are highlighted in black. The phosphopantetheine attachment site is underlined and designated as block ‘I’. The potential DGAT motif is underlined and designated as block ‘II’ and ‘V’. The putative thiolase acyl-enzyme intermediate signature is underlined and designated as block ‘III’. The putative Tyr kinase phosphorylation site is underlined and designated as block ‘IV’. The fatty acid binding protein signature is underlined and designated as block ‘VI’. The putative catalytic active site is underlined and designated as block ‘VII’.

**Figure 3:**
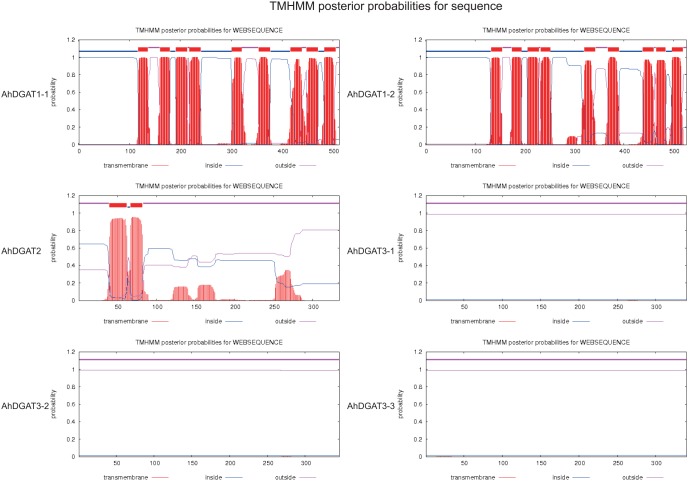
Predicted transmembrane domain for peanut DGAT1, DGAT2 and DGAT3 sequences. The TMHMM web tools (http://www.cbs.dtu.dk/services/TMHMM-2.0/) plot the probability of the ALDH sequence forming a transmembrane helix (0–1.2 on the y-axis). The regions predicted to form transmembrane helix (or helices) are shown in red, while regions of all sequences predicted to be located inside or outside the membrane are shown in blue and pink, respectively. Nine predicted transmembrane helices were identified for AhDGAT1-1 and AhDGAT1-2 sequences, while two transmembrane helix were observed for AhDGAT2 sequences. No transmembrane helice was identified for AhDGAT3-1, AhDGAT3-2 and AhDGAT3-3 sequences.

An analysis of the deduced amino acid sequences of the AhDGATs revealed a number of possible functional domains that are consistent with the substrate utilization properties of the enzyme (Figs. 1 and 2). A protein analysis using the TMHMM Server predicted nine transmembrane domains for AhDGAT1-1 and AhDGAT1-2, which is consistent with the integral membrane enzymes (Fig. 3). This finding is consistent with the nine transmembrane domains predicted for a mammalian DGAT [29], as well as the *Arabidopsis* DGAT1 [30], *B*. *napus* DGAT1, castor DGAT, and soybean DGAT [31], [32], [33]. Other plant DGAT1 proteins also contain multiple transmembrane domains, including eight for tobacco DGAT and 10 for tung tree DGAT1 [8].

An analysis using the Pfam program showed AhDGAT1 proteins belonged to the membrane bound O-transferase (MBOAT) protein family. An MBOAT domain was identified in AhDGAT1-1 (aa 278–499) and AhDGAT1-2 (aa 234–513). This domain is possibly involved in acyl transfer [7].The region spanning R^102^-S^115^HAGLF-K^141^ in AhDGAT1-1 (K^116^-S^129^HAGLF-K^155^ in AhDGAT1-2) is highly conserved amongst other DGAT1s and contains the putative acyl-CoA binding signature spanning residues R^102^-G^118^ (R^116^-G^132^ in AhDGAT1-2), as well as the putative active site catalytic residues R^133^LIIEN^138^ (R^147^LIIEN^152^ in AhDGAT1-2) [34]. An 81 bp insertional mutation (a repeat of exon 2) in the *Arabidopsis* DGAT1 (AtDGAT1) gene resulted in a 27 aa tandem repeat in this DGAT1 region of mutant AS11 [34], which led to a decrease in the seed oil content [35]. The final four residues of the acyl-CoA binding signature of AhDGAT1-1, S^115^HAG^118^ (S^129^HAG^132^ in AhDGAT1-2), are also the first four residues in the AS11 tandem repeat. This correlation is a strong indication of the importance of this motif for activity [34].

As reported previously [34], a putative DAG/phorbol ester binding motif, HKW-XX-RH-X-Y-X-P, a signature sequence observed to be unique to DGAT but absent in ACATs [36], is present in the AtDGAT1 sequence at 414–424 aa. In the AhDGAT1 sequence, this putative DAG/phorbol ester binding motif is found within a highly conserved interface of a near-amphiphilic/highly hydrophobic region extending from residues 405 to 415 in AhDGAT1-1 and from residues 419 to 429 in AhDGAT1-2. A visual examination of AhDGAT1-1 also revealed the sequence L^182^A^183^-X-H^185^-X-X-X-P^189^-X-X-X-V^193^ (L^196^-A^197^-X-R^199^-X-X-X-S^203^-X-X-X-I^207^ in AhDGAT1-2). Such motifs have been identified as targets of the sucrose non-fermenting-related protein kinase 1(SnRK1) family, which may be involved in the global regulation of carbon metabolism [37]. First identified in AtDGAT1 [34], similar motifs are now recognized in other plant DGAT1 sequences.

There is a phosphopantetheine attachment site spanning residues G^143^ to M^164^ in AhDGAT1-1 (G^157^ to M^178^ in AhDGAT1-2). A putative thiolase acyl-enzyme intermediate binding motif, previously cited in the *Arabidopsis* sequence by Zou et al. (1999) [34], is also found in AhDGAT1. It contains an invariant Pro208 at the N-terminus of this motif in AhDGAT1-1 (Pro222 in AhDGAT1-2). This proline is thought to participate in presenting the fatty acyl group to the active site for esterification to (diacyl) glycerol [38]. There is also a fatty acid binding protein signature spanning residues A^373^ to N^389^ in AhDGAT1-1 (A^387^ to N^403^ in AhDGAT1-2) [34], which contains a putative tyrosine phosphorylation site: Y^384^ (Y^398^ in AhDGAT1-2).

The two AhDGAT1 proteins also contain one potential N-linked glycosylation site (N-X-S/T), which is also present in GmDGAT1-2, GmDGAT1-3, and PvDGAT1-1, but not in AtDGAT1, GmDGAT1-1, or PvDGAT1-2 (Fig. 1). AhDGAT1-1 contains a leucine zipper motif with signature residues at L213, L220, L227 and L234. The presence of the putative C-terminal ER retrieval motif is also detected in AhDGAT1s (–YYHDL–, YYHDW–) and was found in other plant DGAT1s (tobacco DGAT1, –YYHDV–; *Arabidopsis* DGAT1; and castor DGAT1, –YYHDL–). These putative ER retrieval motifs (–Φ–X–X–K/R/D/E–Φ–COOH, where Φ is any large hydrophobic amino acid residue) are positioned at the extreme C-termini and very likely serve as general ER localization signals [39].

Hydropathy plots of the predicted protein indicated the absence of any transmembrane domains in AhDGAT3-3 (Fig. 3), which is consistent with that of AhDGAT3-1 and AhDGAT3-2. The absence of the signal sequence also confirms its cytosolic nature. When the deduced amino acid sequence of AhDGAT3-3 was examined for a number of structural motifs, potential DGAT motifs at H^54^VRYYGD^59^ and H^200^KIMELFSRNND^211^ (Fig. 2) were identified, which matched reported members of the acyltransferase family. The insertion of a few amino acids between critical His and Asp residues has also been reported in *Mycobacterium tuberculosis* bifunctional wax ester synthase/DGAT [40]. An alignment with known acyltransferases also confirmed the presence of the DGAT catalytic motif present in cytosolic peanut DGAT at R^329^FLGEN^334^. In addition, a putative Tyr kinase phosphorylation site P^177^KAETMIY^184^ is found in AhDGAT3-3. There are several protein kinase C and casein kinase 2 phosphorylation sites present in the protein. There is a phosphopantetheine attachment site between residues G^24^GGGCVSVPVRLRK^38^. A putative thiolase acyl-enzyme intermediate signature is also found in AhDGAT3-3 at T^135^NPDCESSSSSSESES^150^. An invariant Pro is present in the hydrophobic block at position 33 in AhDGAT3-3 between a putative phosphopantetheine attachment site and a thiolase acyl enzyme intermediate signature, and thus, may be responsible for presenting the fatty acyl group to the active site for esterification to the glycerol backbone. The fatty acid binding protein signature pattern is present between residues K^249^SGSIALLQEFERVVGAEG^267^.

### Phylogenetic analysis

**Figure 4:**
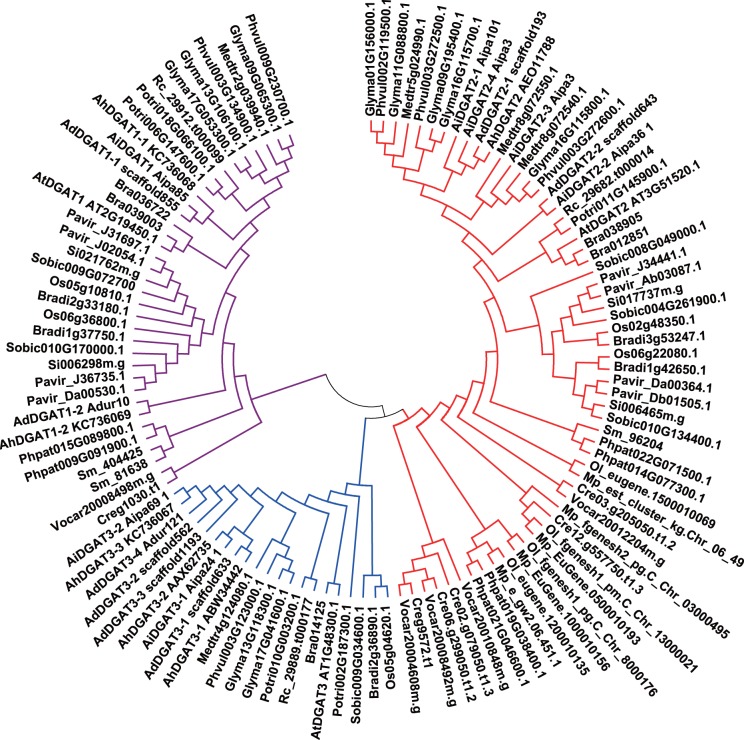
Phylogenetic tree of DGAT1, DGAT2 and DGAT3 gene families reconstructed by the neighbor-joining (NJ) method. Gene sequences other than peanut DGATs were shown by their nomenclatures found of www.phytozome.org, with the abbreviations. Colored branches indicated different groups of proteins. Red: DGAT2, blue: DGAT3, purple: DGAT1. Bootstrapping with 1,000 replicates was used to establish the confidence limits of the tree branches.

To examine the relationships among different sources of DGAT genes, sequences from representative eukaryotic species belonging to plant monocotyledons (*Oryza sativa*, *Brachypodium distachyon*, *Sorghum bicolor*, *Setaria italica*, *Panicum virgatum*), eudicots (*Arabidopsis thaliana*, *Brassica napus*, *Ricinus comunis*, *Populus trichocarpa*, *Medicago truncatula*, *Glycine max*, *Arachis duranensis*, *Arachis ipaensis*, *Phaseolus vulgaris*, *Brassica rapa*), fern (*Selaginella moellendorfii*), moss (*Physcomitrella patens*), algae (*Chlamydomonas reinhardtii*, *Volvox carteri*, *Ostreococcus lucimarinus*, *Micromonas pusilla* RCC299), were selected to construct the phylogenetic tree by the neighbor-joining method (Fig. 4).

All but the two picoplankton species, *M. pusilla* and *O. lucimarinus*, possess DGAT1. The DGAT2 gene is present in all the genomes examined here. The DGAT3 gene is identified in all the genomes examined with the exception of plant monocotyledons (*S. bicolor* and *P. vulgaris*), fern (*S. moellendorfii*), moss (*P. patens*), and algae (*C. reinhardtii*, *V. carteri*, *O. lucimarinus*, and *M. pusilla* RCC299).

As shown in the phylogenetic tree, all of the DGAT protein family members clustered into three major clades: the DGAT1 clade, DGAT2 clade, and DGAT3 clade. The AhDGAT1-1 and AhDGAT1-2 proteins were grouped with DGAT1 enzymes from eudicots and were separate from those of mosses, monocotyledons, and green algae. The DGAT2 protein family fell into six main groups: monocot DGAT2, dicot DGAT2, moss DGAT2, plant-like algal DGAT2, animal-like algal DGAT2 and algal DGAT2. The AhDGAT2 protein was more closely related to the type-2 DGATs from eudicots. The DGAT3 protein family was divided into two distinct subfamilies. AhDGAT3-1, AhDGAT3-2, and AhDGAT3-3 were grouped with DGAT3s from eudicots, separate from those of monocotyledons.

### Tissue-specific expression patterns

**Figure 5:**
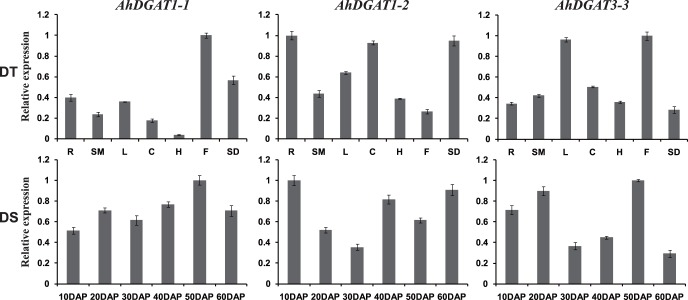
Expression analysis of three *AhDGAT* genes using qRT-PCR in seven peanut tissues and at six stages of seed development. DT (different tissues): R, root; SM, stem; L, leaf; C, cotyledons; H, hypocotyls; F, flower; SD, seed. DS (10 to 60 DAP): six developmental stages of seeds. The relative mRNA abundance was normalized with respect to the peanut *AhACT11* gene. The bars were standard deviations (SD) of three technical repeats.

qRT-PCR was used to confirm the expression patterns of the three novel genes in different peanut tissues and at different stages of seed development. The alpha actin 11 (*AhACT11*) gene was used as an internal reference control for total RNA input [41]. As shown in Fig. 5, these three genes displayed specific temporal and spatial expression patterns across different tissues and developmental stages. *AhDGAT1-1* showed higher transcript abundance in flowers and seeds than in any of the other tissues tested. Levels of *AhDGAT1-2* transcripts were higher in roots, seeds, and cotyledons, followed by leaves, stems, and hypocotyls, with the lowest levels in flowers. The highest abundance of *AhDGAT3-3* transcripts was in flowers and leaves and the lowest was in seeds.

The expression patterns of three *DGAT* genes across six developmental stages of seeds are also shown in Fig. 5. The *AhDGAT1-1* transcript remained relatively low during the initial stage of seed development but increased gradually during later stages of seed development, peaking at 50 DAP, and decreased thereafter until 60 DAP. Levels of the *AhDGAT1-2* transcript were highest at 10 DAP and decreased dramatically thereafter. At 40 DAP, *AhDGAT1-2* transcript levels began increasing again, with higher expression levels through 60 DAP. The *AhDGAT3-3* gene showed higher expression levels at 20 and 50 DAPs and much lower levels during the other stages.

### Expression patterns of *AhDGATs* in peanut under abiotic stress

**Figure 6:**
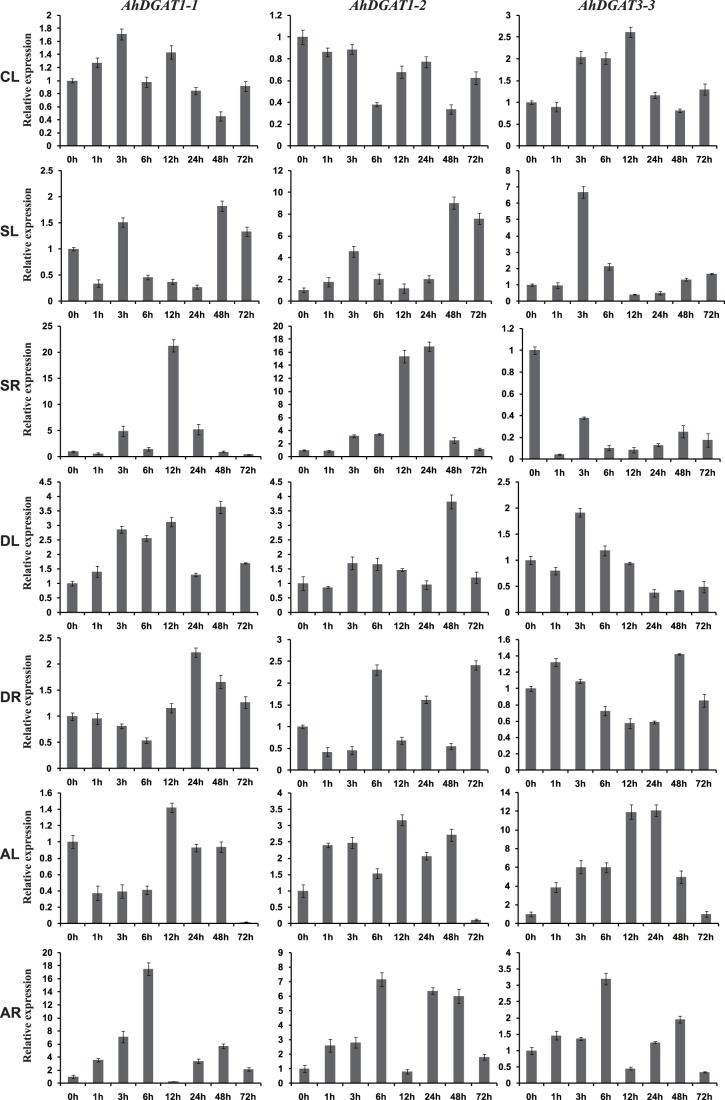
Expression analysis of three *AhDGAT* genes using qRT-PCR under different stresses. CL (0 h to 72 h), leaves exposed to cold (4°C) treatment. SL (0 h to 48 h), leaves exposed to high salt (200 mM NaCl) treatment. SR (0 h to 72 h), roots exposed to high salt (200 mM NaCl) treatment. DL (0 h to 72 h), leaves exposed to 20% PEG-6000 treatment. DR (0 h to 72 h), roots exposed to 20% PEG-6000 treatment. AL (0 h to 72 h), leaves exposed to 100 uM ABA treatment. AR (0 h to 72 h), roots exposed to 100 uM ABA treatment. The relative mRNA abundance was normalized with respect to the peanut *AhACT11* gene. The bars were standard deviations (SD) of three technical repeats.

To confirm the expression patterns of three *DGAT* genes under cold, salt, drought and ABA stresses, we monitored changes of these transcripts in peanut leaves and roots. Figure 6 shows the expression patterns of three *DGAT* genes in peanut leaves upon cold treatment. The expression levels of *AhDGAT1-1* and *AhDGAT3-3* increased under cold stress, peaking at 3 h and 12 h, respectively, and then decreased. The expression of *AhDGAT1-2* gradually decreased under cold stress. After 72 h, the level of the *AhDGAT1-2* transcript remained lower than in untreated leaves.

The expression patterns of *AhDGATs* in peanut leaves and roots after treatment with 200 mM NaCl were also monitored (Fig. 6). The expression patterns of *AhDGAT1-1* and *AhDGAT3-3* were different in leaves and roots. There was no obvious change in the levels of the *AhDGAT1-1* transcript in peanut leaves following salt treatment, although levels of the *AhDGAT1-1* transcript in roots were higher 12 h after salt treatment, increasing nearly 21-fold. The transcript levels of *AhDGAT1-2* increased in both leaves and roots under salt stress, with peak expression levels of 9-fold greater at 48 h in leaves and 16-fold greater at 24 h in roots compared with non-treated controls. Transcript levels of *AhDGAT3-3* decreased rapidly from 1 h to 72 h in roots of seedlings subjected to the salt treatment, but increased in leaves after 3 h of treatment, increasing nearly 7-fold.

A 20% solution of PEG-6000 was used to mimic drought stress to monitor the expression patterns of *AhDGATs* in peanut leaves and roots (Fig. 6). The expression levels of *AhDGAT1-1* increased under drought stress, with a peak level of 3.6-fold greater at 48 h in leaves and 2-fold greater at 24 h in roots compared with non-treated controls. The expression of *AhDGAT1-2* in leaves increased with a peak level of 3.8-fold greater at 48 h under the drought treatment. In roots, the expression of *AhDGAT1-2* increased slightly 6 h after treatment, and then decreased from 6 h to 48 h. After 72 h, *AhDGAT1-2* transcripts reached a maximum level, with the greatest increase being approximately 2.4-fold. There was no obvious change in the abundances of *AhDGAT3-3* transcripts in peanut leaves or roots after drought treatment.

We also examined the response of *AhDGAT* genes to exogenously applied ABA, which is a plant signaling molecule involved in plant defense signaling pathways (Fig. 6). There was no obvious change in the levels of the *AhDGAT1-1* transcript in peanut leaves following ABA treatment, although levels of the *AhDGAT1-1* transcript in roots were higher 6 h after initial exposure to exogenous ABA. The greatest increase was about 17.5-fold in roots. The expression levels of *AhDGAT1-2* and *AhDGAT3-3* increased after ABA treatment, peaking at 12 h and 24 h, respectively, with increases of approximately 3-fold and 12-fold, respectively. Levels of *AhDGAT1-2* and *AhDGAT3-3* transcripts increased in roots, where they reached their maximum levels 6 h after ABA treatment, with increases of approximately 7- and 3-fold, respectively.

### Heterologous expression of *AhDGATs* in the yeast TAG deficient mutant

**Figure 7:**
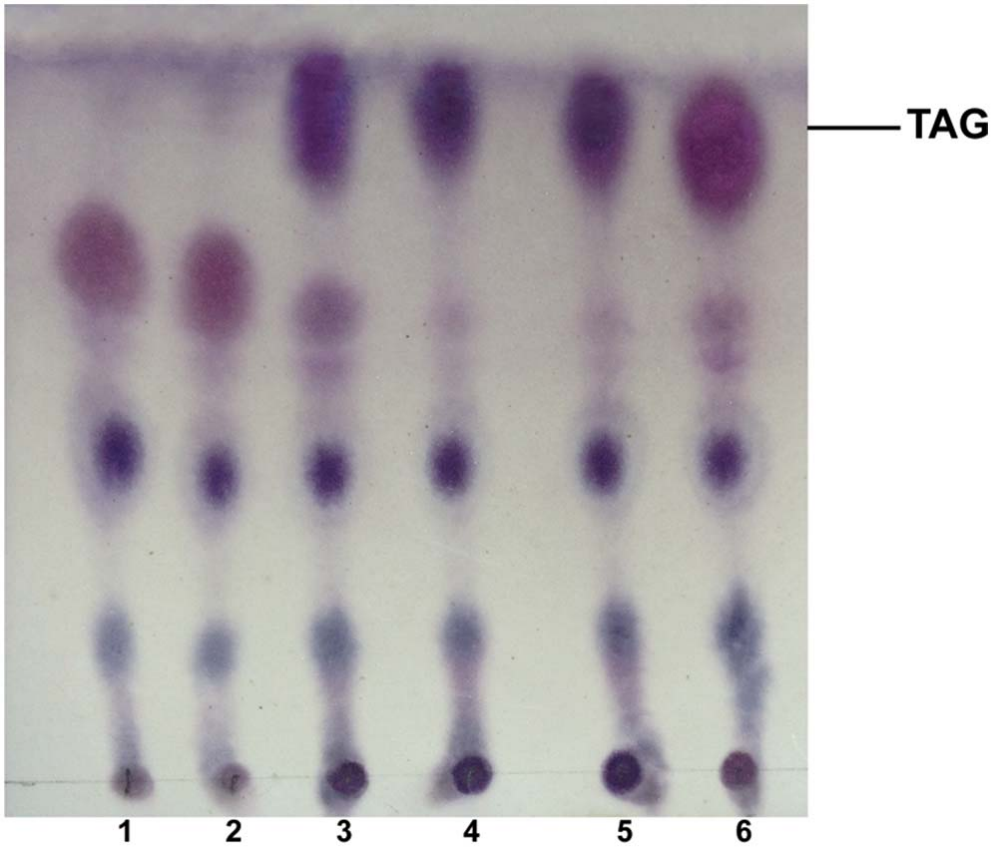
Evaluation of TAG biosynthesis in the yeast quadruple mutant (H1246) complemented with *AhDGAT* genes. Lipid extracts from the yeast cells were separated by TLC and lipid spots were visualized as described in Materials and Methods. The neutral lipid-deficient quadruple mutant strain H1246 (1) and the mutant harboring the empty vector (pYES2) (2) were used as the negative controls. The wild-type strain INVSc1 was used as a the positive control (3). The quadruple mutant expressing *AhDGAT1-1* (4), *AhDGAT1-2* (5) and *AhDGAT3-3* (6) was analyzed.

In the yeast *S. cerevisiae*, four genes, *DGA1* (DGAT2 homolog), *LRO1* (encoding an enzyme that catalyzes the phospholipid:diacylglycerol acyltransferase reaction), *ARE1* and *ARE2* (both involved in steryl ester synthesis) were found to contribute to TAG synthesis [42]. A quadruple disrupted strain with combined deletions in these four genes is devoid of TAG and lacks lipid bodies [19]. To verify whether *AhDGAT1-1*, *AhDGAT1-2* and *AhDGAT3-3* indeed encode proteins with DGAT activity, the coding sequences of these putative *DGAT* genes were expressed individually in the TAG deficient *S. cerevisiae* quadruple mutant strain (H1246) [43]. INVSc1 was used as the positive control. The empty vector pYES2 was transformed into the mutant strain as the negative control. Following expression, the yeast cells in the late stationary phase of growth were used for the extraction of total lipids. TLC of total lipids revealed that TAG was undetectable in the quadruple mutant strain carrying the empty expression vector, whereas upon expression of three *AhDGAT* genes a prominent spot corresponding to TAG appeared (Fig. 7).

**Figure 8:**
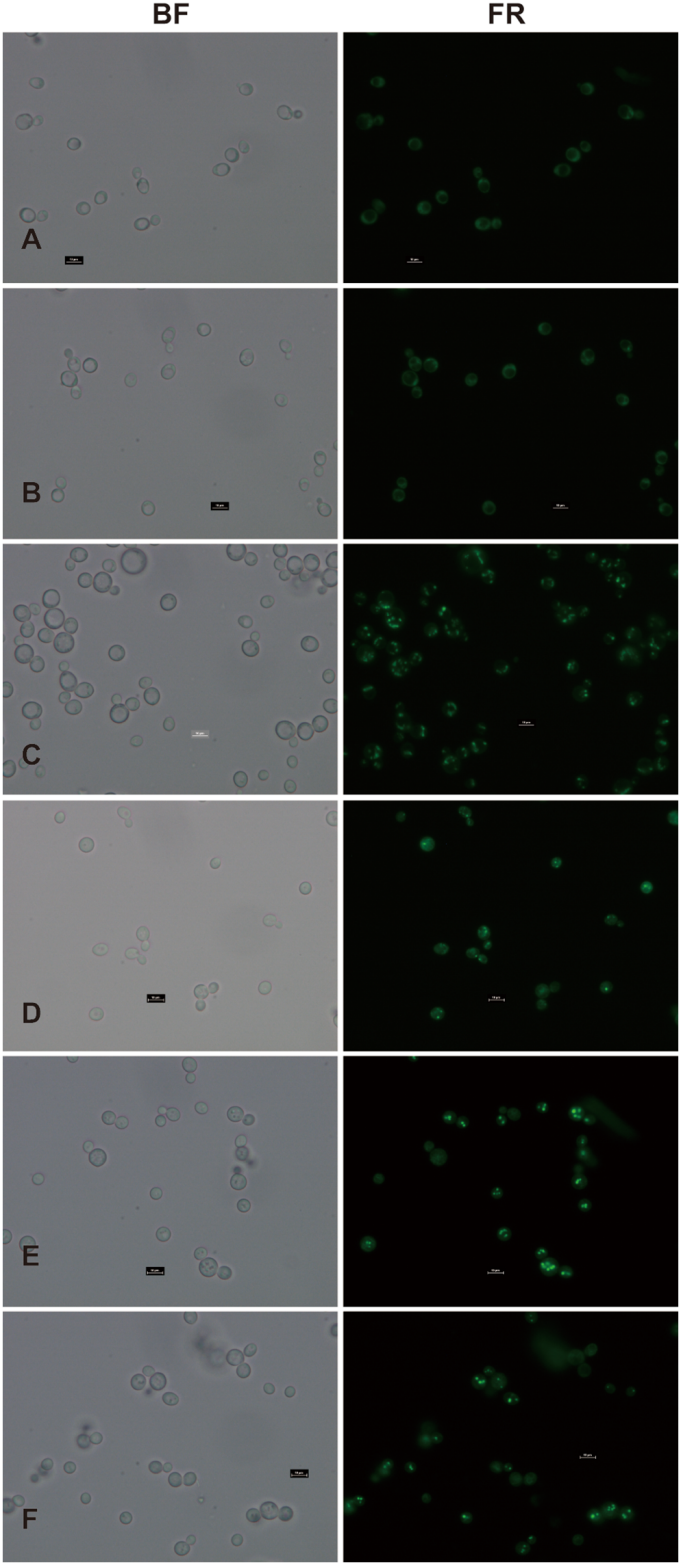
Lipid body formation is restored upon expression of *AhDGATs* in the yeast strain H1246. Neutral lipid accumulation in lipid bodies was visualized in yeast cells with the fluorescent dye BODIPY505/515. The neutral lipid-deficient quadruple mutant strain H1246 (A) and the mutant harboring the empty vector (pYES2) (B) were used as the negative controls. The wild-type strain INVSc1 was used as a the positive control (C). The quadruple mutant expressing *AhDGAT1-1* (D), *AhDGAT1-2* (E) and *AhDGAT3-3* (F) was analyzed. BF, Bright-field images; FR, images of BODIPY505/515 fluorescence.

In yeast cells, storage lipids mainly accumulate during the stationary growth phase in the form of TAGs and steryl esters to lipid bodies, which can be visualized using the fluorescent dye BODIPY505/515. To visualize the lipid bodies generated by the expression of these three genes, the yeast transformants were stained with BODIPY505/515 (Fig. 8). We found that whereas lipid bodies were absent in the mutant or the mutant transformed with empty vector (pYES2), they were abundantly present in wild type and in the mutant strain transformed with three *AhDGAT* genes. These results suggest that expression of three *AhDGAT* genes in the quadruple mutant strain can completely restore its ability to form lipid bodies through interactions with the yeast lipid synthesis pathway, which is consistent with the results of TLC.

### Fatty acid profile of yeast TAG deficient mutant expressing *AhDGATs*

**Figure 9:**
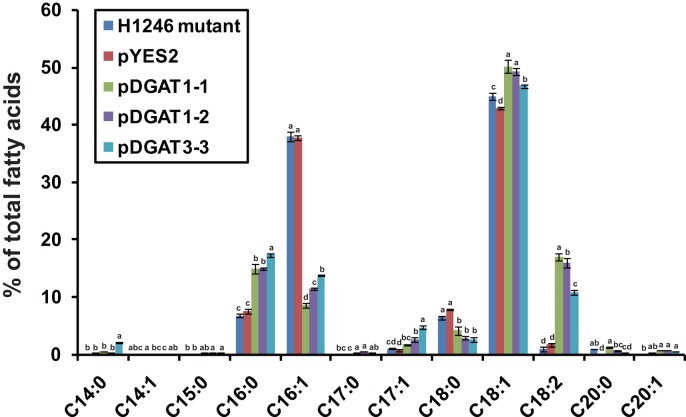
Impact of *AhDGATs* expression on fatty acid profiles of yeast. The bars were standard deviations (SD) of three technical repeats. For the same fatty acid component, numbers with different letters are statistically significant (P<0.05).

We investigated whether the expression of *AhDGATs* has any effects on the fatty acid composition of cellular lipids. As shown in Fig. 9, the expression of three *AhDGAT* genes in the quadruple mutant resulted in differential fatty acid composition of lipids compared with the mutant strain or the strain transformed with empty vector (pYES2). The observed tendency was an evident decrease of fatty acids C16∶1 and C18∶0, and a significant increase in C16∶0, C18∶1, and C18∶2. In the *AhDGATs*-transformed quadruple mutant, C16∶1 and C18∶0 were decreased by 64%–77% and 35–60%, respectively, compared with that of the non-transgenic control strain. In contrast, the expression of *AhDGATs* in the quadruple mutant led to a more than one-fold increase in C16∶0, and up to a 15-fold increase in C18∶2 when compared with those of the control strain. The C18∶1 was increased by 4%–11% compared with that of the control strain. Furthermore, the transformants with *AhDGAT1-1*, *AhDGAT1-2* and *AhDGAT3-3* showed significant differences in the production of some fatty acids, suggesting that these three enzymes have slightly different functions in peanut plants. These results suggest that the expression of *AhDGATs* in yeast can increase the incorporation and transfer of endogenous unsaturated fatty acids into lipids.

## Discussion

### Characterization of the peanut DGAT genes

It has long been understood that DGAT enzymes play important roles in TAG biosynthesis. DGAT is the only enzyme in the pathway that is thought to be exclusively committed to TAG synthesis, and thus it is considered a key enzyme in this reaction [1]. *A. hypogaea* (peanut, Fabaceae) is one of the most economically-important oil-producing crops, and two types of DGAT genes have been identified from peanut. Saha et al. (2006) identified a soluble DGAT3 (AhDGAT3-1) from immature peanut cotyledons and expressed its full length in *Escherichia coli*, where the recombinant protein had high levels of DGAT activity but no wax ester synthase activity [9]. Peng et al. (2013) identified two isozymes of DGAT2 in peanut and expressed both of them as full-length recombinant proteins in *E. coli*. The total fatty acid levels of the AhDGAT2a-GST and AhDGAT2a-GST transformants, as well as levels of C12∶0, C14∶0, C16∶0, C16∶1, C18∶1n9c and C18∶3n3 fatty acids, increased markedly, whereas C15∶0 and C21∶0 levels were lower than in non-transformed cells and those containing empty-vectors [28]. In this study, three genes that likely encode DGAT proteins were isolated from peanut. They are two DGAT1 genes and one novel DGAT3 gene. Analysis of the deduced amino acid sequence of the AhDGATs revealed a number of possible functional domains that were consistent with the substrate utilization properties of the enzyme. A protein hydrophobicity analysis predicted that nine putative transmembrane helices were identified in the AhDGAT1-1 and AhDGAT1-2 sequences; however, no transmembrane helix was observed in AhDGAT3-3.

We surveyed the putative DGAT1, DGAT2 and DGAT3 gene families in eukaryotes, discovering that most of them are present in the eukaryotes searched. All but the two picoplankton species, *M. pusilla* and *O. lucimarinus*, possess DGAT1. For these two picoplankton species, the absence of a gene encoding DGAT1 could again be due to gaps in the genomic sequence; it is also conceivable that their DGAT1s are so divergent that they cannot be identified via sequence similarity searches. However, there is a precedent for the possibility that they truly do not possess a DGAT1. The yeast (*S. cereviseae* and *Candida albicans*) and Basidiomycetes fungi (*Laccaria bicolor*, *Schizophillum commune* and *Agaricus bisporus*) also do not encode DGAT1, although *S. cerevisiae* has two genes with some sequence similarity to DGAT1, ARE1 and ARE2 [30]. These are sterol:acyl-CoA acyltransferases, which have partial DGAT activity and may act as the DGAT1 equivalents in yeast [42]. However, no such sequences were identified in the picoplankton species listed above.

The DGAT2 gene is present in all the genomes examined. In contrast to most of higher plants and mosses, which encode single DGAT2 genes, algal species seem to have multiple genes for putative DGAT2s. The vast majority of algal DGAT2s seem to be distantly related to both higher plant and animal DGAT2s. It has been proposed that the various putative DGAT2 isoforms found in modern algal groups could represent a very ancient gene duplication event that occurred prior to the subsequent divergence of various eukaryotic lineages. These isoforms were then gradually lost in eukaryotic lineages that formed the basal groups of complex multicellular organisms until only one particular isoform was selected prior to the speciation of multicellular organisms [44], [45].

It is now clear that the DGAT1 and DGAT2 families arose from different ancestors during the emergence of eukaryotes, and they followed convergent evolution in eukaryotes despite having evolved separately since the early eukaryotes [30], [44]. The DGAT3 enzymes are phylogenetically divergent from DGAT1 and DGAT2, and little is known about its evolutionary origins. At first, we surveyed the putative DGAT3 genes from the representative genomes of *Viridiplantae*. We found that this gene was not identified in the genomes of two plant monocotyledons, nor the mosses and algae examined. But it is not sure if this gene exists in other lineages of eukaryotes. Thus, we conducted a much more detailed survey of fully sequenced genomes for the presence of DGAT3 homologs in eukaryotes, including representatives of *Amoebozoa*, *Viridiplantae*, *Rhodophyta*, *Heterokonta* (*stramenopiles*), *Haptophyta*, *Fungi*, *Cryptophyta*, *Rhizaria*, *Choanozoa*, *Heterolobosea*, and *Metazoa* (Table S1 and Fig. S2). We detected that DGAT3 gene only exists in *Viridiplantae*, but was not present in the genomes of two plant monocotyledons (*S. bicolor* and P. *vulgaris*), one eudicot (*Cucumis sativus*), one moss (*P. patens*), one fern (*S. moellendorfii*), and nine algae (*C. reinhardtii*, *V. carteri*, *O. lucimarinus*, *O*. *tauri*, *Ostreococcus sp. RCC809*, *M*. *pusilla* RCC299, *M*. *pusilla CCMP1545*, *Chlorella variabilis NC64A*, *Coccomyxa subellipsoidea C-169*). Thus it is inferred that DGAT3 could only exist in plant monocotyledons and eudicots, but not in moss and algal species in *Viridiplantae*, which suggests that DGAT3s might evolve in the last common ancestor of the *Viridiplantae*.

### Functional analysis of the peanut DGAT genes

*DGATs* from several plant species have been studied and their expression levels are regulated in a tissue-specific and time-dependent manner. Our results indicated that the transcript abundance of *AhDGAT1-1* was higher in flowers and seeds than in other tissues examined, whereas the *AhDGAT1-2* transcript was more abundant in roots, seeds, and cotyledons. It has been reported that *AtDAGT1* gene was expressed in a wide range of tissues but most strongly in developing embryos and flower petals [6], showing a similar expression pattern to *AhDGAT1-1*. In *R. communis*, there was little difference in the steady state expression of *RcDGAT1* between leaves and developing seeds, and the highest expression of *RcDGAT1* was at 10 DAP (days after pollination) [46]. In *V. galamensis*, transcript levels of *VgDGAT1* were much higher in embryos (sampled at 20 DAP) than in roots, stems, leaves, or pericarp. During seed development, *VgDGAT1* transcripts moderately increased at early stages (from 10 to 17 DAP) and then sharply rose to a peak at 24 DAP. Subsequently, *VgDGAT1* expression dropped gradually until 45 DAP [22]. The *AtDGAT3* gene was highly expressed in cork, xylem, hypocotyl, and senescent leaf, and moderately expressed in pollen and stem [47]. *AtDGAT3* was shown to be ubiquitously expressed in various developmental stages and highly (>2-fold) expressed in the early stages of seed development [47]. The peanut *AhDGAT3-1* mRNA was detected only in immature seeds between 8 to 14 d after flowering (DAF) and 15 to 24 DAF. At the third stage of seed development (between 25 and 30 DAF), the transcript was barely detectable and no transcript was detected in late stage of seed development, leaf, and root. But the *AhDGAT3-3* gene showed different expression patterns. *AhDGAT3-3* showed higher transcript abundance in flowers and leaves, followed by cotyledons and stems, with the lowest levels in seeds. The *AhDGAT3-3* gene also showed elevated expression levels at the initial two stages of seed development, but the highest level was seen at 50 DAP. Thus, the same type of *DGAT* genes from different plants may have different spatial and temporal expression patterns, which requires further investigation.

Plant growth and yield are strongly influenced by abiotic stresses, such as drought, salt and cold. Plants respond and adapt to these conditions through an array of biochemical and physiological changes [48], [49]. Biological membranes are the first barrier that separates cells from their environment and are a primary target for damage during environmental stress. Many organisms have developed mechanisms to maintain the appropriate fluidity of membrane lipids. These mechanisms include changes in the proportions of types of lipid and alterations in the lipid/protein ratio [50]. For example, the most widely recognized change in cell membranes at low temperatures is the unsaturation of lipid acyl chains [51], [52]. Glycerolipids with unsaturated fatty acids have a lower melting point and more flexibility than glycerolipids with saturated acyl chains [53]. Our results indicated that *AhDGAT1-2* was distinctly enhanced under all stress treatments except for cold-stressed leaves. The expression of *AhDGAT1-1* increased in all materials after stress treatments except for cold-, salt- and ABA-treated leaves, whereas transcript levels of *AhDGAT3-3* increased in cold- and salt-stressed leaves, ABA-treated leaves and roots. Thus, we infer that these *AhDGAT* genes, which incorporate unsaturated or saturated fatty acids to defined positions in glycerolipids, may be involved in regulating some abiotic stresses in peanut.

Heterologous expression studies in the *S. cerevisiae* TAG-deficient quadruple mutant strain H1246 confirmed that *AhDGATs* encode functional proteins, restoring TAG biosynthesis and lipid body formation. By comparing fatty acid profiles of lipids produced by the quadruple mutant expressing *AhDGATs* and the control strain, we found that three *AhDGAT* genes could preferentially incorporate unsaturated C18 fatty acids into lipids from yeast cells. In *O. tauri*, *P*. *tricornutum*, *Thalassiosira pseudonana*, *C. reinhardtiithe*, and *Tropaeolum majus*, functions of DGAT2 or DGAT1 were also confirmed by restoring TAG biosynthesis in this quadruple mutant of *S. cerevisiae* [23], [24], [34], [54], [55].

In conclusion, three novel DGAT-like genes from peanut were cloned. In a yeast expression system, these three genes restored TAG and lipid body formation, and favored incorporation of unsaturated fatty acids into TAGs, which has potential value in the genetic engineering of peanut for a high oil content or other special characteristics. It has been reported that both acyl-CoA dependent and acyl-CoA independent mechanisms make contribution to TAG synthesis in plants [3] and yeast [56]. We also isolated the genes encoding putative phospholipid:glycerol acyltransferases (PDAT) from peanut, which catalyze the transfer of an acyl group from the sn-2 position of phosphatidylcholine to the sn-3 position of diacylglycerol, yielding TAG and sn-1 lyso-phosphatidylcholine [3]. Further research is needed to determine the contribution of PDAT-like protein as well as the newly identified AhDGATs to the overall production of TAG in peanut.

## Materials and Methods

### Ethics Statement

No specific permits were required for the described field studies. No specific permissions were required for these locations and activities. The location is not privately-owned or protected in any way and the field studies did not involve endangered or protected species.

### Plant materials

Peanut plants (*A. hypogaea* L. cultivar Huayu 19) were grown in a growth chamber with a 16 h light/8 h dark photoperiod at 26°C/22°C day/night temperatures. Leaves, stems, cotyledons, hypocotyls, and roots were sampled from the seedlings at the trefoil leaf stage. Seeds were sampled at 10, 20, 30, 40, 50, and 60 days after pegging (DAP). Flowers were collected when the seedlings were in the flowering phase. For the cold treatment, seedlings in the soil at the trefoil leaf stage were kept at 4°C, and leaves were sampled separately either before cold treatment (0 h) or after continuous exposure to 4°C for 1, 3, 6, 12, 24, 48, or 72 h. For stress treatments, roots of seedlings grown in soil were flushed carefully with tap water to remove all soil, and then submerged in solutions of 200 mM NaCl, 20% PEG-6000, or 100 µM ABA. Leaves and roots were sampled separately after treatment for 0, 1, 3, 6, 12, 24, 48, or 72 h. All samples were immediately frozen in liquid nitrogen and stored at –80°C until required.

### Identification of DGAT family genes in a peanut cDNA library using Bioedit software

The cDNA sequences used in this study came from three cDNA libraries from three institutes (data not shown). That is Shandong Peanut Research Institute, Oil Crops Research Institute of Chinese Academy of Agricultural Sciences, and Crops Research Institute of Guangdong Academy of Agricultural Sciences. All expressed sequence tags (ESTs) of the 36,741 cDNA sequences were saved in the FASTA format. The amino acid sequences of DGAT genes of *Arabidopsis*, *AtDGAT1* (At2G19450), *AtDGAT2* (At3G51520) and *AtDAGT3* (At1G48300), were used as query to search for homologous genes from the peanut cDNA library. Before searching for members of the *DGAT* gene family, a local nucleotide database file was created using Bioedit software. A local BLAST procedure was then run to find the homologous genes of the *DGAT* family. Using this method, we found three genes that may encode DGAT proteins.

### Isolation of full-length cDNA sequences

Total RNA was extracted using the RNeasy Plant Mini kit (Qiagen, Valencia, CA, USA). Contamination with genomic DNA was eliminated by treatment with recombinant DNase I (Qiagen), as recommended by the vendor. Only RNA preparations having an A260/A280 ratio of 1.8–2.0 and an A260/A230 ratio >2.0 were used for subsequent analysis. The integrity of RNA was verified by electrophoresis through 2% agarose gels, followed by SYBR Green staining. First-strand cDNA synthesis was carried out with 2 µg RNA using an RT-PCR kit (Promega, Madison, WI, USA) according to the manufacturer’s procedure.

We performed PCR with the LA PCR system (Takara, Dalian, China), using 2.5 µl of 10×PCR buffer with MgCl_2_, 1 µl of each primer (10 µM) (Table S2), 4.0 µl of 10 mM dNTPs, 1 µl of cDNA sample, 0.5 µl of LA Taq DNA polymerase, and 15 µl of double-distilled water. The PCR products were separated by electrophoresis through a 1% agarose gel, and purified using a Gel Extraction Kit (Takara) according to the manufacturer’s protocol. The purified products were then cloned into the pMD18-T Easy vector (Takara) and sequenced (Shangon, Shanghai, China).

### Sequence analyses

The open reading frames (ORFs) and encoded amino acid sequences of all genes were deduced using BioXM 2.6. Physicochemical properties of the deduced proteins were predicted using Protparam (http://www.expasy.ch/tools/protparam.html). Active sites of the protein sequences were analyzed by comparisons against the PROSITE database. Predicted transmembrane domains in DGAT proteins were identified using the TMHMM Server (version 2.0) (http://www.cbs.dtu.dk/services/TMHMM) and visual inspection. The putative subcellular localizations of the candidate proteins were estimated by TargetP (http://www.cbs.dtu.dk/services/TargetP/) and Predotar (http://urgi.versailles.inra.fr/predotar/predotar.html).

### Phylogenetic analysis

Homologs of each member of the *Arabidopsis* DGAT family were identified by BLASTP searches with datasets from Phytozome v9.1 (www.phytozome.net) and Peanut Genome Project (http://peanutbase.org/home). Our group has also sequenced and analyzed the genome of *Arachis duranensis*, and the data has not been published. The *DGAT* genes were also identified from our draft genome of *Arachis duranensis*. Only those sequences with an e-value less than e−^50^ were considered as members of the DGAT family. In each tree, gene sequences other than peanut DGATs were displayed using the nomenclaturewith the following abbreviations: At, *Arabidopsis thaliana*; Glyma, *Glycine max Wm82.a2.v1*; Medtr, *Medicago truncatula*; Phvul, *Phaseolus vulgaris*; Ai, *Arachis ipaensis*; Ad, *Arachis duranensis*; Rc, *Ricinus communis*; Potri, *Populus trichocarpa*; Bra, *Brassica rapa chiifu-401 v1.2*; Os, *Oryza sativa*; Bradi, *Brachypodium distachyon*; Sobic, *Sorghum bicolor v2.1*; Si, *Setaria italica*; Pavir, *Panicum virgatum v1.1*; Phpat, *Physcomitrella patens v3.0*; Sm, *Selaginella moellendorffii*; Cre, *Chlamydomonas reinhardtii*; Vocar, *Volvox carteri*; Ol, *Ostreococcus lucimarinus*; Mp, *Micromonas pusilla RCC299*. Table S3 provides a detailed description of the proteins used and the corresponding accession numbers. Amino acid sequences were aligned using the ClustalX program with the implanted BioEdit [57]. The neighbor-joining (NJ) method in MEGA4 [58] was used to construct the phylogenetic tree. Bootstrapping with 1,000 replicates was used to establish the confidence limits of the tree branches. Default program parameters were used.

### Quantitative real-time RT-PCR (qRT-PCR)

qRT-PCR analysis was performed using a LightCycler 2.0 instrument system (Roche, Germany). The action 11 gene (*AhACT11*) was selected as the reference gene [41]. Three pairs of gene-specific primers (Table 1) were designed after analyses of the target genes’ sequences. qRT-PCR reactions were performed using the SYBR Premix Ex Taq polymerase (Takara) according to the manufacturer’s instructions. Each 20-µl reaction was comprised of 2 µl of template, 10 µl of 2× SYBR Premix, and 0.4 µl (200 nM) of each primer. The reactions were subjected to an initial denaturation step of 95°C/10 s, followed by 40 cycles of 95°C/5 s, 60°C/30 s and 72°C/10 s. A melting curve analysis was performed at the end of the PCR run over the 60–95°C range, increasing the temperature stepwise by 0.5°C every 10 s. The baseline and quantification cycle (CP) were automatically determined using the LightCycler Software. Zero template controls were included for each primer pair, and each PCR reaction was carried out in triplicate. The relative quantification method (delta-delta Cp) was used to evaluate quantitative variation.

### Heterologous expression of *AhDGATs* in yeast

The *AhDGATs* in the pYES2 plasmid were transformed into the yeast quadruple mutant H1246MATa (*dga1Δ lroΔ are1Δ are2Δ*), which is deficient in oil synthesis, using the polyethylene glycol/lithium acetate method according to the manual (Invitrogen, Carlsbad, CA, USA). The auxotrophic *S. cerevisiae* strain INVSc1 (MATa his3-Δ1 leu2 trp1-289 ura3-52) was used as the positive control. Yeast cells transformed with an empty pYES2 plasmid were used as the negative control. Yeast transformants were selected by growth on synthetic complete medium lacking uracil (SC-ura), supplemented with 2% (w/v) glucose. The colonies were transferred into liquid SC-ura with 2% (w/v) glucose and grown at 28°C overnight. The overnight cultures were diluted to A = 0.4 in induction medium (SC-ura+2% galactose+1% raffinose) and were induced by incubating at 28°C overnight [59]. Cells were harvested by centrifugation, washed three times with double-distilled water and used for the extraction of total lipids.

### 4,4-difluoro-1,3,5,7-tetramethyl-4-bora-3a,4a-diaza-s-indacene (BODIPY505/515) staining and microscopy

The BODIPY505/515 staining method described by Mou et al. (2011) was used to visualize the intracellular lipid bodies as an indicator of TAG formation [60]. The lipophilic fluorescent dye BODIPY505/515 was purchased from Invitrogen and dissolved in anhydrous dimethyl sulfoxide to achieve a 10 mM stock solution, which was stored at −20°C. For yeast cell staining, a 1 mL suspension of yeast cells in the culture medium was stained with 0.2 µL of 10 mM BODIPY 505/515, to achieve the final concentration of 2 µM, for 1 min at room temperature. After yeast cells were stained with BODIPY505/515, a Nikon Eclipse 80i microscope with a blue light (488 nm) as the excitation wavelength was used to image and quantify lipid bodies in yeast cells. A Nikon CCD DS-file digital camera was used to capture the images.

### Lipid extraction and analysis

For the extraction of total lipids from yeast, harvested cell pellets were lyophilized, ground with a mortar and then added to a conical flask with 7 mL methanol/chloroform (2∶1, v/v). The solution was placed in an ultrasound bath (28 kHz, 600W) for 10 min at 50°C. After transferring the lipid extract to a fresh tube, tissues were re-extracted with 1.5 mL methanol/chloroform (2∶1, v/v). This operation was repeated two times. Lipid extracts were combined, and 2.5 mL chloroform and 3 mL NaCl (1%, w/v) were added. The samples were vortexed, centrifuged, and the upper phases were discarded. The organic phase (lower phase) was transferred to a fresh glass tube. The combined organic phases were dried under N_2_ and dissolved in hexane. TAGs were separated from total lipids by thin layer chromatography (TLC) using a solvent system of hexane/ether/acetic acid (70∶30∶1, v/v/v) [61]. Individual lipid spots were visualized by exposing the silica gel plates (Qingdao, China) to the vapor of anisaldehyde/acetic acid/sulfuric acid (1∶100∶2, v/v/v).

Total fatty acids were extracted and transmethylated with methanolic HCl from yeast cells according to Browse et al (1986) [62]. All samples were analyzed using a 7890A/5975C gas chromatography (Agilent Technologies, California, USA) equipped with a 5975C single quadrupole GC/MSD detector and an HP-INNOWAX capillary column (30 m×250 µm×0.25 µm). High purity nitrogen was used as the carrier gas. Measurements were performed using peak height area integrals expressed as a percentage of the total of all integrals. The experiment was carried out in triplicate, and the data subjected to analysis of variance using DPS software (Zhejiang University, China) Version 7.05. Duncan’s multiple range test was employed to determine the statistical significance (P<0.05) of the differences between the means.

## Supporting Information

Figure S1 **Pairwise identity comparison of the DGATs from different sources.**
(EPS)

Figure S2 **Phylogenetic tree of DGAT3 gene family reconstructed by the neighbor-joining (NJ) method.**
(EPS)

Table S1 **Genome-wide survey of the DGAT3 enzymes from different organisms.** The table shows the kingdoms, species, gene names and accession numbers of the sequences detected in the analyses.
(XLS)

Table S2 **DNA sequences of oligonucleotide primers used in this study.**
(DOC)

Table S3 **The DGAT1, DGAT2 and DGAT3 enzymes used for the phylogenetic analyses.** The table shows the kingdoms, species, gene names and accession numbers of the sequences used in the analyses.
(XLS)
