# Supplementary material for: Cloning and Functional Analysis of Three Diacylglycerol Acyltransferase Genes from Peanut (Arachis hypogaea L.)
Source: PLoS One. 2014 Sep 2;9(9):e105834. doi: 10.1371/journal.pone.0105834 (PMC4152018; doi:10.1371/journal.pone.0105834)
Supplement: Table S2 — DNA sequences of oligonucleotide primers used in this study. (DOC) [file pone.0105834.s004.doc]

Table S2. DNA sequences of oligonucleotide primers used in this study

| Name | Oligonucleotide sequence 5’–3’ |
| --- | --- |
| Full-length cDNA sequence cloning | |
| DGAT1-1-F | ATGGCGATTTACCAGAGCGT |
| DGAT1-1-R | TTATTCAGTTTTGACCTTCCTA |
| DGAT1-2-F | ATGGCGATTTCCGATGTG |
| DGAT1-2-R | TTAGTTATGTTCCCTGTGCC |
| DGAT3-3-F | ATGGAGGTTTCCGGCACC |
| DGAT3-3-R | CTATTCATTAGTACTTTCCTGATT |
| Real-time RT-PCR | |
| qDGAT1-1-F | CATCAACTTCACTTCTGTATC |
| qDGAT1-1-R | GTAATCCTTCTACCTTCTCAT |
| qDGAT1-2-F | TCTAAGAATGCTGCTGTATT |
| qDGAT1-2-R | AGGAAGTTAGTGACAATGG |
| qDGAT3-3-F | CCGCTGAAGTTTTGATGAAG |
| qDGAT3-3-R | CCTTTGGTGGGGAATCAGCT |
| qACT11-F | TTGGAATGGGTCAGAAGGATGC |
| qACT11-R | AGTGGTGCCTCAGTAAGAAGC |
| Vector construction | |
| vDGAT1-1-F | TAGGATCCATGGCGATTTACCAGAG |
| vDGAT1-1-R | CACCTCGAGTTATTCAGTTTTGACCTT |
| vDGAT1-2-F | TAGGATCCATGGCGATTTCCGATGT |
| vDGAT1-2-R | CACCTCGAGTTAGTTATGTTCCCTGTG |
| vDGAT3-3-F | TAGGATCCATGGAGGTTTCCGGCA |
| vDGAT3-3-R | CACCTCGAGCTATTCATTAGTACTTTC |
